# The Effects of a Low Calorie Ketogenic Diet on Glycaemic Control Variables in Hyperinsulinemic Overweight/Obese Females

**DOI:** 10.3390/nu12061854

**Published:** 2020-06-22

**Authors:** Małgorzata Magdalena Michalczyk, Grzegorz Klonek, Adam Maszczyk, Adam Zajac

**Affiliations:** 1 Department of Sport Nutrition, Academy of Physical Education in Katowice, 72a Mikołowska Street, 40-065 Katowice, Poland; a.maszczyk@awf.katowice.pl (A.M.); a.zajac@awf.katowice.pl (A.Z.); 2 Lenacor Diet Clinic, Świętojańska 2a Street, 41-400 Mysłowice, Poland; klonek.grzegorz@gmail.com

**Keywords:** low-calorie ketogenic diet (LCKD), adult, hyperglycaemia, hyperinsulinemia, IR, HDL-C

## Abstract

Diet is a factor which can influence both glycaemic variables and body mass. The aim of this study was to compare the influence of a 12-week, well-planned, low-calorie ketogenic diet (LCKD) on hyperglycaemic, hyperinsulinemic and lipid profile in adult, overweight or obese females. Ninety-one females who participated in the study were divided into two groups: a LCKD group who followed a hypocaloric ketogenic diet (8% of carbohydrate, 72% of fat and 20% of proteins) (*n* = 46), and a control group (CG) (*n* = 45) who continued their typical diet (50% of carbohydrates, 32% of fat and 18% of proteins). Methods: Baseline and post-intervention glucose (Gl), insulin (I), glycated haemoglobin (HbA1c), Homeostatic model assessment HOMA-IR, triglycerides (TG) and high-density cholesterol (HDL-C) were evaluated. Also, body mass (BM), waist circumference (WC), hip circumference (HC) and thigh circumference (TC) were measured. Results: Compared with the CG, there were significant changes observed in the LCKD group regarding all biochemical variables. Also, BM, TC, WC and AC changed significantly in the LCKD group compared with the CG. Conclusions: The 12-week LCKD intervention changed the glucose control variables, body mass, as well as waist, hip and thigh circumferences. A low-calorie ketogenic diet may be recommended for adult females with glucose control variables disturbance and excess body mass.

## 1. Introduction

All over the world, the number of people who are diagnosed with fasting hyperglycaemia and hyperinsulinemia increases each year [1]. This condition implies insulin resistance (IR) phenomenon and finally results in type 2 diabetes mellitus (T2DM) [1,2,3,4]. IR is triggered by a lack of response from glucose transporters to insulin in muscle, heart and adipose tissue. [5,6]. A primary feature of IR is excessive hepatic glycogenolysis and gluconeogenesis, even when insulin is elevated [6,7,8,9]. Permanent high blood glucose concentration promotes the glycation processes in various proteins, such as haemoglobin, collagen and others [8]. As a result, these proteins are stiff, brittle, less soluble and less susceptible to repair processes. In consequence, protein glycation causes significant organ damage, leading to nephropathy, encephalopathy or retinopathy [4]. The combination of IR and compensatory hyperinsulinemia increases the dyslipidaemia [8,9] characterised by a high plasma triglyceride (TG) and low high-density lipoprotein cholesterol (HDL-C) concentration [10]. Additionally, there is substantial evidence for a correlation between insulin resistance/hyperinsulinemia and sleep-related breathing disorders, excessive somnolence after a meal, weight gain, mainly in the abdomen area, and difficulty losing weight, frequent feeling of hunger and increased appetite for sweets [11]. Moreover, most people with hyperinsulinemia suffer from high body mass which can contribute to other health problems, such as hypertension, atherosclerosis or cancer [3,5,12,13].

In order to lower blood glucose levels or insulin concentration and simultaneously reduce body mass, different strategies are promoted, such as diet change, supplementation, increased physical activity, pharmacological therapy or surgery [3,10,14,15,16]. It seems logical that the reduction of the amount of carbohydrates in the diet should result in a lower glucose and insulin postprandial increase. Additionally, as a consequence of lower glucose supply, weight reduction will occur [3]. Currently, many prominent scientists have confirmed that diets recommending a limited intake of carbohydrates reduce glucose and insulin concentration as well as body mass [17,18,19,20,21,22,23,24]. Carbohydrate-restricted diets have become a popular strategy not only within the scientific community, but also among doctors and dietitians [2,3,25]. One variation of a carbohydrate restriction diet is the ketogenic diet (KD) [12,14]. The KD is a high-fat, low-carbohydrate diet. It consists of up to 70% of fat and below 50 g of carbohydrate of the total daily calorie intake [12,14]. Traditional KD was initiated using a specified ketogenic ratio which is defined as the ratio of grams of fat to grams of carbohydrate plus protein [26]. Higher ratios result in greater degrees of ketosis. Recent work has shown that lower ketogenic ratio diets are frequently as efficacious as higher ones [27,28]. The main intention of the KD is to limit glucose intake as much as possible [14,18]. In such conditions, in most cells, the secretion of lipolysis enzymes increases, which boosts energy production from fats stored in the adipose tissue as well as those supplied with the diet. In turn, the central nervous system cells and the red blood cells do not have enzymatic mechanisms to oxidase fat, therefore they use fat oxidation metabolites—ketone bodies (KB) acetoacetate and β-hydroxybutyrate—as a source of energy [12,17,29]. Following the ketogenic diet results in an increased production of KB and, in addition to KB, glucose synthesized in the liver from glycerol derived from lipolysis and amino acids supplied with the diet can be used as an energy substrate.

Although the KD has been used for almost 100 years to treat epilepsy [26,27,28,30], in recent years, it has been studied as part of the treatment for other diseases such as type 2 diabetes (T2DM), obesity [20,25,29], metabolic syndrome, cardiovascular problems [22,25,31,32] and others [18,33]. There is supportive evidence that the use of the KD in obese patients with IR is effective in improving glycaemic control variables [25,34,35]. In our study, we compared the effects of a 12-week KD with a typical western diet (control group, CG) on fasting glucose, insulin, haemoglobin A1c, HOMA-IR (Homeostasis Model Assessment-Insulin Resistance), HDL-C and TG concentration in overweight and obese females. In addition, we evaluated the impact of the KD on body mass (BM) and waist, hip and thigh circumferences.

## 2. Material and Methods

### 2.1. Study Sample

**Figure 1:**
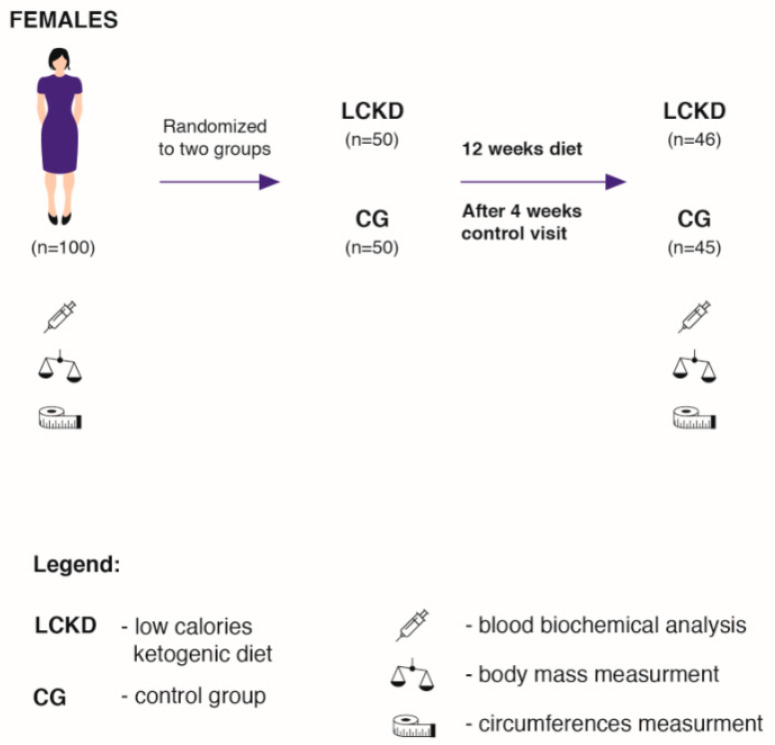
Scheme of the descriptive analysis. LCKD—low calories ketogenic diet; CG—control group.

The study was conducted in cooperation with the Lenacor Diet Clinic in Mysłowice. The subjects were recruited from a group of females (body mass index (BMI) > 25.0 kg/m^2^) who reported to the clinic independently between April 2019 and October 2019. The clinic has been operating since 2015 and is managed by qualified dietitians. One hundred adult females (Figure 1) were randomly assigned to two groups: the low-calorie ketogenic diet group (LCKD, *n* = 50), and to a control group (CG, *n* = 50). The randomisation was performed in several stages. Initially, 300 volunteers enrolled for the study (first degree, *n* = 300), out of whom, every third person was randomly selected for the study. This group was again randomly divided into two study groups (second degree; *n* = 2 × 50). Randomisation of the first degree consists of a random selection of people or cases to be examined. For second-degree randomisation, we used the sealed envelopes method. In this stage, during their first visit in the laboratory, to be more specific, every subject drew an envelope containing the prescribed diet. Consequently, two groups—LCKD and CG—were formed.

In addition to following specific dietary requirements, the LCKD group consumed 20% fewer calories daily than the total daily energy expenditure (TDEE). The subjects in the CG consumed the same diet as before joining the experiment. For the purpose of the study, the CG did not change the macronutrient proportions or energy intake. Ninety-one subjects completed the study. Four subjects from the LCKD group and five from the CG resigned from the study due to personal reasons (Figure 1). The individuals from the LCKD group were not able to maintain a calorie-restricted diet and consumed fast foods, sweets and alcohol, which were not included in the prescribed diet. The subjects from the CG did not come to control visits without providing any explanations. The inclusion criteria were as follows: age between 30 and 60, glucose > 5.5 mmol/L, insulin > 10 uU/mL, not following any kind of diet or food elimination in the last 12 months and mild exercise more than twice a week. The exclusion criteria were as follows: the intake of any supplements with an established lipid and glucose profile, energy expenditure through physical activity > 1000 kcal/week, insulin resistance therapy, dyslipidaemia therapy, hypertension (systolic blood pressure > 140 mmHg and/or diastolic blood pressure > 90 mmHg or antihypertensive treatment), multiple allergies, celiac disease or other intestinal diseases, any condition that could limit the mobility of the subject and make laboratory visits difficult, life-threatening diseases or conditions that could affect adherence to the measurements or treatments, vegetarianism or a need for other specific diets and alcoholism or other drug addiction.

Before the experiment began, all subjects were informed about the study objectives and the accompanying risks and benefits. They were also informed about the possibility of withdrawing from the experiment at any time. All subjects read and signed the informed consent to participate in the study. The study protocol was approved by the local Ethics Committee at the Academy of Physical Education in Katowice, Poland (ethical reference KB-5/2015).

### 2.2. Dietary Procedures

The dietary intervention lasted 12 weeks (Figure 1). A tailor-made hypocaloric KD was prescribed for each subject, where daily caloric consumption was 20% less than total daily energy expenditure (TDEE). The TDEE was calculated according to the commonly accepted model: (TDEE = Activity Factor (AF) × Resting Metabolite Rate (RMR)) [2]. RMR was measured at the beginning of the experiment by means of a metabolic cart, MetaLyzer 3B (Cortex, Leipzig, Germany), in the Human Performance Laboratory at the Academy of Physical Education in Katowice. AF was determined based on available indicators for adults with low physical activity (AF = 1.4) [36]. Additionally, before the experiment, the subjects were asked to take home and complete a 72 h food diary (two weekdays and one weekend day) [3,10]. The dietary records were evaluated by a nutritionist in order to assess previous eating habits and daily caloric consumption. Before the experiment, all subjects consumed a typical Western-style diet [3,10,37]. This type of diet is characterized by a high intake of saturated fats, simple sugars and salt [10,37]. The respondents consumed fast foods, alcohol, sweetened beverages and sweets. The diet consumed by all subjects before the experiment consisted of >50% of carbohydrates, <20% of protein and >30% of fat. They had never experimented with calorie restriction diets or with restricted carbohydrate diets before. The diet composition was analysed using DIETETYK 6.0 software (Jumar, Poland).

### 2.3. Diet Composition

The composition of the diets is shown in Table 1. LCKD meals were prepared in the form of 24 h menus for all seven days of the week—2 main meals during the day between 6.00 am and 8.00 pm. The LCKD was composed of high-quality food products [3]. The subjects consumed healthy fats, mainly monounsaturated fatty acids from olive oil, dairy products and nuts. The LCKD also contained n-6 and n-3 polyunsaturated fatty acids in a ratio of 5–6:1. The diet included fish, such as mackerel, herring and sardines, which are rich in n-3 fatty acids, combined with meat and eggs, providing high-quality protein. The protein consumption was 1 g/per kg of body mass, which was a reference value for adults [38]. Ketogenic ratio in our diet was 1:1.5. LCKD meals consisted of poultry, fish, beef, veal and lamb, dried beef, chopped meet tartare, carpaccio, cured ham, eggs and cheese, olive oil, butter as well as raw green vegetables without limitation [3]. Hot beverages were restricted to tea and coffee without sugar or herbal extracts. The foods and drinks that the subjects avoided included alcohol and sugar. Dairy products—sweetened milk, fruit yogurt, whole grain or white bread, pasta, white rice, sweets, instant tea or barley coffee were completely prohibited. Trans fats (margarines and their derivatives) and processed meats with added sugar were not allowed. No more than two cups of coffee and at least 3 litres of water were recommended each day. Artificial sweeteners were not allowed (saccharin, cyclamate, acesulfame, aspartame and sucralose).

The CG consumed their usual diet, labelled the conventional diet, which was the modern Western-style diet [3]. The subjects from CG consumed 3–4 main meals daily as well as 1–2 snacks. This diet is characterised by high-caloric intake of energy-dense foods, saturated and n-6 fatty acids, and refined sugars, excessive salt and alcohol intake and low consumption of n-3 fatty acids and fibre [3,37]. The Western-style diet is based on fast food, sweetened beverages, sweets and alcohol [4]. The diet consisted of mainly white flour products (bread, bagels and pasta), white rice, potatoes, beef, pork, sausages, poultry, cheese, fried eggs, vegetables, fruit, margarine, sunflower oil, whole milk, coffee with milk and sugar, tea, fruit juices, carbonated drinks, such as cola, and water. The subjects consumed medium- to high-glycaemic-index snacks (buns, chocolate bars, bananas) and meals (white rice, potatoes, white bread, pasta).

Supplements: The subjects in the LCKD group were given 2 tablets of a multi-vitamin mineral supplement (Centrum, Pfizer Corporation Austria Gesellschaft m. b. H, Wiedeń, Austria), each tablet of the multi-vitamin-mineral supplement contained: vitamin A 800 mcg, Beta-carotene 200 mcg, vitamin D 5 mcg, vitamin E 10 mg, vitamin C 60 mg, vitamin B1 1.4 mg, vitamin B2 1.6 mg, vitamin B6 2 mg, folic acid 200 mcg, vitamin B12 1 mcg, niacin 18 mg, biotin 150 mcg, pantothenic acid 6 mg, vitamin K 30 mcg, calcium 162 mg, potassium 40 mg, phosphorus 125 mcg, iron 14 mg, magnesium 100 mg, cupper 700 mcg, zinc 750 mcg, manganese 250 mcg, iodine 150 mcg, molybdenum 25 mcg, chlorine 36.3 mg, chromium 25 mcg, selenium 25 mcg. Additionally, they consumed one tablet of vitamin D_3_ 2000 international unit (IU) and one tablet of calcium carbonate 1500 mg.

### 2.4. Diet Control

The LCKD was well planned. The quality and quantity of food products used to prepare the meals were strictly defined to maintain proper proportions between all the major macronutrients. During the 12 weeks of the experiment, the subjects received daily menus. They were given a detailed list of products and their weight as well as a recipe for preparation. The subjects prepared the meals by themselves. Each of them was given a detailed list containing foods permitted and prohibited in the LCKD, which gave them the necessary knowledge about how to control their diet composition. They analysed their diet during follow-up visits after every 4 weeks of the experiment. Additionally, for better diet control, once a week, in the morning before a meal, the subjects measured the level of ketone bodies in the blood using either a glucometer (Optimum Xido or Optimum Xido Neo, U.S.A. Abbott Laboratories, Chicago, Illinois) with the function of measuring ketone bodies or commercially available urine reagent strips (Ketur-Test, Roche Diagnostics, Basel, Switzerland). The KB’s level results were discussed with the dietitian during follow-up visits.

### 2.5. Experimental Design

Before and after twelve weeks of the experiment, in both the LCKD group and the CG, fasting blood evaluations and somatic measures were carried out to determine anthropometric and biochemical variables.

### 2.6. Biochemical Analysis

Before and after the study, the following biochemical variables were evaluated: glucose (Gl, mmol/L), insulin (I, μg/dL), HbA1c (mg/L), triglycerides (TG, mg/dL) Beckman Coulter, high-density lipoprotein cholesterol (HDL-C, mg/dL), using Beckman Coulter diagnostic kits (Gl-OSR6221, I-OSR 33410, Variant II Turbo HbA1c Kit 2.0 270-2455EX, TG-OSR66118, HDL-Cholesterol OSR6687). β-hydroxybutyrate (β-HGB-mmol/L) was measured using Randox UK diagnostic kits (Ranbut) and HOMA-IR was calculated by the formula: fasting glucose concentration (mmol/L) multiplied by the plasma insulin concentration and divided by 22.5 [39].

### 2.7. Body Mass and Circumference Measurements

Before and after twelve weeks of the experiment, in both the LCKD group (*n* = 46) and the CG (*n* = 45), BM, BMI, waist circumference (WC), hip circumferences (HC) and thigh circumferences (TC) were measured.

The evaluation of BM was performed by multifrequency bioimpedance analysis (MF-BIA) using the InBody 220 (Biospace Co., Ltd., Seoul, Korea). BMI was calculated using the following formula (BMI = body mass (kg)/height (m^2^)). WC was measured in the standing position at approximately 0.5 cm of the midpoint between the lowest rib and the iliac crest. HC was measured at the level of hip bones. TC was measured 2 cm below the umbilicus. The measurements were taken under laboratory conditions, according to the instructions of the manufacturer using an anthropometric tape. During the anthropometric evaluations, the subjects wore underwear only.

### 2.8. Statistical Analysis

Body mass, body composition and biochemical variables are expressed as mean ± standard deviation (SD). Before using a parametric test, the assumption of normality was verified using the Kolmogorov–Smirnov test. Two-way analysis of variance (ANOVA) was used with significance set at *p* < 0.05 to determine statistically significant differences between intra- and inter-groups. When appropriate, a Tukey’s post hoc test was used to compare selected data, and the effect size (eta-squared; η^2^) of each test was calculated to determine the significance of the results. The effect size (η^2^) was classified according to Hopkins as 0.01—small, 0.06—medium and 0.14—large [40]. The remaining analyses were performed using STATISTICA (Stat Soft, Inc. (2018) version 12, StatSoft Polska Sp. z o.o., Kraszewskiego 36 Street, 30-110 Kraków, Poland).

## 3. Results

Ninety-one females (in the LCKD group—46: age—42 ± 7; height—165 ± 6 cm, and in the CG 45: age—41 ± 6; height—165 ± 4 cm) participated in the study. Table 2 and Table 3 present the intra-group and the inter-group baseline and post-intervention results of glucose control variables (Gl, I, HbA1c and HOMA-IR), TG, HDL- C, β- HB, BM and body circumference.

### Biochemical Variables

The intra-group differences in post-intervention of Gl, I, HbA1c, HOMA-IR, TG and HDL-C were observed compared with baseline values in the LCKD group. A lower post-intervention concentration of I (F = 88,00, *p* = 0.001, η^2^ = 0.21), Gl (F = 57,92, *p* = 0.001, η^2^ = 0.18) and HbA1c (F = 7, 52, *p* = 0.007, η^2^ = 0.08) TG (F = 52, 90, *p* = 0.001, η^2^ = 0.19) and β-HB (F = 890, 39, *p* = 0.001, η^2^ = 0.33) (Table 2), and higher post-intervention concentration of HDL-C (F = 152, 29, *p* = 0.001, η^2^ = 0.25) were also observed (Table 2). HOMA-IR value was also lower (F = 121, 02, *p* = 0.001, η^2^ = 0.22). After the 12-week dietary intervention, in the CG group, there were no statistically significant intra-group differences of Gl, I, HbA1c, HOMA-IR, TG or HDL-C compared with baseline (Table 2).

The inter-group ANOVA analysis revealed lower post-intervention values of I (F = 134, 36 *p* = 0.001, η^2^ = 0.24), Gl (F = 24, 69, *p* = 0.001, η^2^ = 0.11), HbA1c (F = 9, 82, *p* = 0.002, η^2^ = 0.09), TG (F = 47, 88, *p* = 0.001, η^2^ = 0.18), HOMA-IR (F = 145, 91 *p* = 0.001, η^2^ = 0.24), β-HB (F = 831, 28 *p* = 0.001, η^2^ = 0.31) and higher HDL-C (F = 146, *p* = 0.001, η^2 =^ 0.24), which were observed after the LCKD, compared with post-intervention values of I, Gl, HbA1c, TG, HDL-C, HOMA-IR and β-HB in the CG.

The effect of the dietary interventions on BM, WC, HC and TC in intra-group measures were revealed. A decrease in post-intervention values of BM (F = 32, 93, *p* = 0.001, η^2^ = 0.07), WC (F = 37, 33, *p* = 0.001, η^2^ = 0.09), HC (F = 40, 51, *p* = 0.001, η^2^ = 0.11) and TC (F = 33, 38, *p* = 0.001, η^2^ = 0.08) after the LCKD were observed. No significant changes in these variables were noted in the CG (Table 2).

The inter-group ANOVA showed lower values of BM after the LCKD intervention (F = 35, 64, *p* = 0.001, η^2^ = 0.08) as well as lower WC (F = 57, 37, *p* = 0.001, η^2^ = 0.16), HC (F = 31, 23, *p* = 0.001, η^2^ = 0.06) and TC (24, 86, *p* = 0.001, η^2^ = 0.06) compared with these values in the CG (Table 3).

## 4. Discussion

For years, the effects of different diets, especially with limited carbohydrate content, have been the focus of our research [3,5,37,41,42]. We examined the impact of low and moderate carbohydrate and low-caloric diets on the blood lipid profile, glucose control variables, weight and body composition in different populations. In the study with carbohydrate restriction, the diet was composed with healthy products containing healthy fats and carbohydrates in order to prevent negative health consequences [3,41,42]. It has been confirmed that diets containing mainly MUFA’s and smaller amounts of SFA’s are associated with lower risks of metabolic syndrome as well as IR and body mass disturbance [43]. The results of this study demonstrate that implementing a low-calorie (with a 20% restriction of calorie intake), very low-carbohydrate, ketogenic diet over a 12-week period in overweight and obese females was effective in controlling glucose variables, the lipid profile variables, body mass and body circumferences. In addition, vitamin and mineral supplementation in the LCKD group could have had an impact on the obtained results. The choice of females as a research group was dictated by two factors. First of all, so far, most studies regarding ketogenic diets have been carried out on male subjects. Secondly, females are much more difficult material in this type of research, and it was a challenge for us. They have “weaker will” and their motivation decreases more often [10]. Undoubtedly, this is due to the different sex hormones in males and females [44]. In males, testosterone has a huge impact on motivation, eagerness to fight and the desire to achieve goals and be successful [45]. These effects may occur via androgen pathways modulating dopaminergic regions, thereby affecting behaviour on longer timescales [45]. On the other hand, female sex hormones, oestradiol and progesterone, whose levels are increased during the luteal phase, have a great impact on the perception of flavours and boost the desire for sweets [46]. Unfortunately, for various reasons, we did not have the opportunity to study the level of hormones and it seems to be another important aspect that should be checked in the future concerning this type of diet.

Compared with the CG, which followed a diet adhering most currently recommended macronutrients content for adults, the LCKD group had lower glucose, insulin and glycated haemoglobin concentration and lost significantly more weight. In addition, despite consuming a high percentage of calories as fat, the subjects in the LCKD group maintained better levels of plasma TG and HDL-C. The subjects recruited for this study were overweight or moderately obese females. As such, they were representative of many Polish overweight and obese females with hyperinsulinemia and hyperglycaemia. The interview conducted before the onset of the experiment showed that the subjects followed a not perfect diet and had low physical activity. According to the American College of Sports Medicine and Word Health Organisation (WHO), for general health, adults should walk a minimum of 10,000 steps per day [47,48]. The subjects failed to walk 10,000 steps per day, which is recommended for adults [10], and did not follow the basic rules of a healthy diet, which is eating 4 to 5 meals a day regularly, every 3–4 h. They declared that eating 4 to 5 meals a day is very difficult. Most of the subjects in the LCKD group were positively surprised after receiving the daily menu which contained only 2 main meals. After the experiment was completed, most of them declared that they would continue this diet, not only for health reasons but because it is very practical in everyday life.

The KD drastically deprives the body of carbohydrates, which has a positive influence on glucose and insulin concentration [3,14,15,49]. In many studies, LCKDs have undoubtedly been proven to be effective, in the short as well as long term, as a tool to decrease hyperglycaemia and hyperinsulinemia [12,14,25,50]. In our study, after 12 weeks of following the LCKD, we observed significant changes in fasting insulin and glucose concentration (Table 2). A decrease in considered variables admittedly resulted from the fact that the LCKD daily carbohydrates consumption was less than 50 g [12]. Except for reduced carbohydrate consumption, the LCKD contained a high amount of EPA and DHA omega-3 fatty acids, which contribute to a decrease of glucose and insulin concentration [51,52]. It has been confirmed that high EPA and DHA concentration in the muscle phospholipid cell membranes is associated with a decrease of glucose control disorders or IR [48]. This result was not observed in the CG. A lack of changes in these variables in the CG was caused probably by a relatively higher daily carbohydrate consumption, but also it is possible through elevated hepatic glycogenolysis and gluconeogenesis from reduced central insulin sensitivity [53]. In the CG, after a meal, which contains approximately 50–100 g of carbohydrates, postprandial hyperglycaemia appears, which increases the risk of IR [22]. In physiological conditions, in response to high-glucose concentration, an active complex insulin-receptor activates a cascade reaction through which glucose transporters—GLUT4, are translocated into the cell membrane, causing passage of glucose into the cells. Unfortunately, after following a conventional diet (Table 2) or a low-fat diet, despite the high insulin blood concentration, glucose is not transported inside the cells and is circulating in the blood [3]. Similar results regarding the effects of the LCKD on glucose and insulin concentration are presented by other authors [8,25,33,35,49,50,51]. Samaha et al. [49] in a randomised study with 126 severely obese subjects (BMI—43 kg/m^2^) in an experiment with a low-carbohydrate ketogenic diet (KD) and a hypocaloric low-fat diet (LFD) achieved significantly better results in serum glucose concentration in the LCKD group compared with the LFD group. Also, Westman et al. [23] in the study with the LCD over a 24-week period in patients with obesity and type 2 diabetes mellitus concluded that the LCD leads to a greater improvement in glycaemic control variables. In another study, Westman et al. [24] confirmed that the ketogenic diet may even be a preferable option in the treatment of T2DM. Hussain et al. [34] confirmed these results.

Extremely low glucose consumption by the subjects in the LCKD group caused a significant reduction of HbA1c (Table 2), which is also used as the main indicator to establish glycaemic control [8]. Similar results were achieved by other authors using a carbohydrate-restricted diet [8,9,50]. Yamada et al. [50] showed that HbA1c levels decreased significantly by as much as 7.9% in the LCD group and by only 2.6% in the calorie-restricted group. Also, Wang et al. [8] reported that after a 3-month LCD, fasting HbA1c levels were reduced in T2DM subjects. They claim that the subjects consumed much less high-glycaemic index foods, and had a much higher intake of nuts, which could help improve hyperglycaemia. Tay et al. [9] compared the impact of a low-carbohydrate, high-fat (LC) diet and a high-carbohydrate, low-fat (HC) diet on glycaemic control and cardiovascular disease risk factors in T2DM. After 52 weeks, using both diets, they achieved a substantial reduction of HbA1c and fasting glucose concentration, but subjects following the LC diet, which was high in MUFA and low in SFA, demonstrated a greater improvement of the blood glucose stability. This confirms that the LCKD can be recommended, in particular for obese people with IR or T2DM [54]. In his study, Husain et al. [34] compared the LCKD with a “standard” hypocaloric diet in patients with overweight and obesity and who were also diagnosed with T2DM. He showed that the LCKD was better at improving glucose, insulin and HbA1c concentration. Additionally, the subjects were able to reduce the antidiabetic therapy.

Doctors or dietitians, when suspecting glucose metabolism disorders such as IR or diabetes, recommend fasting glucose concentration and the oral glucose tolerance tests (OGTT). The OGTT shows the dynamics of glucose absorption from the blood into the tissues [10,29]. Possible disorders indicate a state of insulin resistance or diabetes. Unfortunately, OGTT is quite complicated and requires the participant being in the laboratory for longer than 2 h. For prosaic reasons, it is not practical. Therefore, in assessing IR risk, it is more practical and easier to calculate HOMA-IR [8,13]. HOMA-IR is a mathematical, non-invasive model based on the reciprocal loop theory between the liver and β-cells of the pancreas, which regulate the concentration of glucose and insulin [3,55,56]. The model can be used to evaluate the function of the β-cells of the pancreas as well as the level of IR [37]. HOMA-IR, due to its simple formula, should be a standard diagnostic tool used by doctors and nutritionists who work with overweight and obese patients for quick diagnosis of glucose control disorders [3]. It was observed that after 12 weeks of the experiment, the LCKD group showed a significantly reduced HOMA-IR, from 3.73 ± 1.3 to 1.4 ± 0.6. In the CG, no changes of HOMA-IR were observed (Table 2). Johnstone et al. [48], in a study with seventeen obese non-diabetic subjects, had a significantly lower HOMA-IR and fasting glucose after the LCKD than at baseline. Similar results were achieved by other authors using a carbohydrate-restricted diet [3,8,9,18,49]

As compared with the CG, the LCKD is associated with a greater improvement in some risk factors for coronary heart disease, such as serum HDL-C and TG concentrations [56]. Our subjects consumed high amounts of MUFA and omega-3 (EPA and DHA), but smaller amounts of SFA than in a typical ketogenic diet [3]. Probably, these were the two components which directly influenced the favourable changes [57]. According to Howard et al. [58], high consumption of EPA and DHA reduces the concentration of TG in blood plasma through inhibition of their re-synthesis in the liver and enterocytes up to 30%. EPA and DHA affect at least four transcription factors contributing to endogenous hepatic TG and very low density lipoperotein (VLDL) synthesis. They inhibit the activity of liver X receptors (LRX) and sterol regulatory element binding protein (SREBP-1) and activate the farnesol X receptor (FRX) factor, thus limiting the expression of enzymes responsible for lipogenesis [59]. Also, low consumption of glucose and fructose in the LCKD has a positive effect. Dashti et al. [32], after 56 weeks of the ketogenic diet, observed the same results. Also, Samaha et al. [49], during a 6-month experiment with the LCKD and a hypocaloric LFD in obese patients with dyslipidaemia, achieved similar results. In the Peres-Guisado et al. [60] study, positive results in numerous blood variables after using the LCKD were observed. They used the “Spanish Ketogenic Mediterranean Diet” (SKMD), characterized by high amounts of olive oil, various types of salads, fish and red wine. After a 12-week SKMD, they recorded a significantly higher HDL-C (from 42.81 mg/dL to 58.71 mg/dL) and lower fasting TG (from 232.64 mg/dL to 111.21 mg/dL) and glucose (from 118.57 mg/dL to 90.14 mg/dL).

For several decades, the most popular diet, recommended by dietitians and doctors around the world to reduce body weight, has been a low-calorie diet [3,5,34,37]. This model assumed a drastic cut in daily energy intake, below the RMR [5,34,61]. Despite the positive effects of weight reduction, people who followed such a diet reported a constant feeling of hunger, which made them distracted, nervous and even depressed [5]. Finally, after a few days, they either gave up or started eating snacks, which inhibited body weight reduction. There is a reason why scientists are still looking for an optimum reductive diet, which would have the effect of reducing weight but also did not result in the constant feeling of hunger and physical discomfort [3,5,10,62,63]. In recent years, more attention has been paid not only to the caloric content of the reductive diets but also to their macronutrient compositions [3,41,42]. Scientists compare how the different components of macronutrients in a reducing diet can affect the amount of weight reduction [3,37,63]. Several comparisons of restricted carbohydrate and high-fat diets, such as the present study, show a greater weight loss or a significant decrease in body circumferences and BMI achieved using a diet with limited amount of carbohydrates [3,8,10,18,31,34,41,64,65,66]. Our study confirmed that the LCKD influenced the reduction of body weight, BMI, as well as waist, arm and thigh circumferences (Table 3). The same results are presented in many other studies [3,8,18,24,60]. Paoli et al. [18], in a study with overweight women, after 12 weeks of the LCKD achieved significant reduction of body mass, fat and waist circumferences. Also, Samaha et al. [49], after six months of the LCKD, recorded a significantly greater weight loss (5.8 kg versus 1.9 kg) compared with the LFD. Volek et al. [20], after 50 days of a low-carbohydrate ketogenic diet, achieved significant weight loss in males and females. Also, Peres-Guisado et al. [60], after a 12-week LCKD, observed significant body mass reduction (from 109.79 to 95.86 kg), BMI (from 36.99 kg/m^2^ to 32.42 kg/m^2^) and waist circumference (from 114.01 cm to 98.59 cm).

It should be mentioned that in addition to carbohydrate restriction present in the LCKD, high blood β-HB concentration also had an impact on weight reduction [14]. β-HB is one of the main ketone bodies (KB) produced in the mitochondria in the liver as a fat oxidation product during glucose deficit, which suppresses appetite [14,15,29]. When following the LCKD, the KB’s production increases drastically, causing ketosis [15]. The KB’s concentration in blood or urea is measured to assess whether the subject actually follows the diet. KB are used by tissues, especially central nervous cells, as an alternative source of energy [15]. In the LCKD group, after 12 weeks, β-HB achieved 3.46 ± 0.5 mmol/L, which indicated ketosis and confirmed that the subjects were indeed following the diet. Our study has some limitations. We focused mainly on glucose control variables and body mass reduction. We did not evaluate the effect of the LCKD on other important clinical aspects such as bone health, cardiovascular function, renal function and gastro-intestinal health and microbiota. We did not evaluate a hunger and satiety assessment which would improve the quality of our research. Another limitation of this study is the comparison of isocaloric and hypocaloric diets. Our choice not to change calories’ consumption in the control group was dictated by the fact that even if we had applied a 20% calorie reduction, the participants would still have consumed significant amounts of carbohydrates and would not have achieved ketosis. We were mostly interested in ketosis and its impact on measured parameters. Also, our intention was to check how the composition of macronutrients in the diets affects changes in measured parameters. All these limitations should be acknowledged and considered accordingly in future research, which will undoubtedly improve the quality of work.

## 5. Conclusions

In summary, our study demonstrates the short-term, safety, tolerability and efficacy of a low-calorie ketogenic diet, including multi-vitamin complex supplementations, an interventional weight loss and IR prevention. This nutritional therapy intervention resulted in significant weight loss in all participants, along with marked amelioration of glycaemic control, as compared with a standard diet recommended by WHO or EFSA for obese and IR prevention. The long-term safety and efficacy of the proposed diet nutritional therapy strategy warrants further evaluation.

## Figures and Tables

**Table 1:** Average macronutrients and total energy content of the low-calorie ketogenic diet (LCKD) group and the control group (CG) reported during the study.

Contents	LCKD Mean ± SD	CG Mean ± SD
RMR, kcal TDEE	1616 ± 154 2263 ± 216	1637 ± 142 2292 ± 155
TEI, kcal	1810 ± 173	2170 ± 211
Carbohydrate, %	8	50
Carbohydrate, g	36 ± 3.5	286 ± 32
Fibre, g	16 ± 3	15 ± 5
Proteins, %	20	18
Proteins, g	90.5 ± 9	97.6 ± 9.5
Proteins, g/kg bm/d	1 ± 0.6	1.1 ± 0.8
Fat, %	72	32
Fat, g	145 ± 14	77 ± 7.5
SFA, g	35 ± 4	44 ± 3.5
MUFA, g	65 ± 8	15 ± 2
PUFA, g	45 ± 4	18 ± 2
n-6, g	38 ± 3.5	16 ± 1.5
n-3, g	7 ± 0.5	1.7 ± 0.5
EPA and DHA, g	5.4 ± 0.5	0.8 ± 0.1
n-6/n-3	5.5:1	11:1
Cholesterol, g	543 ± 74	685 ± 58

Note: RMR—Resting Metabolic Rate, TDEE—Total Daily Energy Expenditure, TEI—Total Energy Intake, SFA—Saturated Fatty Acids, MUFA—Monounsaturated Fatty Acids, PUFA—Polyunsaturated Fatty Acids, EPA—Eicosapentaenoic Acids, DHA—Docosahexaenoic Acids, n-3—Omega 3, n-6—Omega 6, kg bm/d—kg of body mass/day, SD—standard deviation.

**Table 2:** Biochemical variables before and after the dietary intervention.

Variables	LCKD, BASELINE Mean ± SD	LCKD, AFTER Mean ± SD	CG, BASELINE Mean ± SD	CG, AFTER Mean ± SD
Gl, mmol/L	5.94 ±0.56	4.74 ± 0.90	5.86 ± 0.39	5.82 ± 0.90
I, µg/Dl	14.12 ± 4.75	6.61 ± 2.63	13.78 ± 4.21	13.80 ± 3.63
HbA1c, mg/L	5.87 ± 0.94	5.38 ± 0.74	5.86 ± 0.60	5.90 ± 0.74
HOMA-IR	3.73 ± 1.2	1.38 ± 2.63	3.57 ± 1.09	3.58 ± 1.13
TG, mg/dL	213.45 ± 63.60	129.13 ± 46.23	210.57 ± 36.45	206.44 ± 50.03
HDL-C, mg/dL	36.71 ± 4.42 0.15 ± 0.03	52.99 ± 7.77 3.46 ± 0.06	44.14 ± 5.07 0.11 ± 0.03	43.01 ± 5.03 0.10 ± 0.09
β-HB, mmol/L				

Note: Gl—glucose; I—insulin; HbA1C—glycated haemoglobin; HOMA-IR—Homeostasis Model Assessment-Insulin Resistance; TG—triglycerides, HDL-C—cholesterol high-density lipoprotein cholesterol, β-HB—β-hydroxybutyrate, LCKD—low-calorie ketogenic diet group, CG—control group.

**Table 3:** Anthropometric variables before and after the dietary intervention.

Parameters	LCKG, BASELINE Mean ± SD	LCKG, AFTER Mean ± SD	CG, BASELINE Mean ± SD	CG, AFTER Mean ± SD
BM, kg	89.08 ± 14.68	75.36 ± 13.47	90.63 ± 11.04	89.86 ± 11.30
BMI, kg/m^2^	32.52 ± 4.50	27.47 ± 3.92	33.21 ± 4.55	32.92 ± 4.55
WC, cm	101.04 ± 11.86	87.34 ± 9.50	102.93 ± 10.32	103.67 ± 9.79
HC, cm	112.82 ± 9.89	101.21 ± 7.42	112.55 ± 8.90	111.36 ± 9.36
TC, cm	64.57 ± 6.36	56,91 ± 6,36	65.26 ± 6.71	64.94 ± 7.02

Note: BM—body mass; BMI—body mass index; WC—waist circumference, HC—hip circumference, TC—thigh circumference, LCKD—low-calorie ketogenic diet group, CG—control group.
